# Extent and patterns of community collaboration in local health departments: An exploratory survey

**DOI:** 10.1186/1756-0500-4-387

**Published:** 2011-10-07

**Authors:** James Studnicki, Elena A Platonova, Chris N Eiechelberger, John W Fisher

**Affiliations:** 1 Department of Public Health Sciences, College of Health and Human Services, University of North Carolina, Charlotte, 9201 University City Blvd, Charlotte, NC, 28223, USA; 2 College of Computing and Informatics, University of North Carolina, Charlotte, 9201 University City Blvd, Charlotte, NC, 28223, USA

## Abstract

**Background:**

Local public health departments (LHDs) in the United States have been encouraged to collaborate with various other community organizations and individuals. Current research suggests that many forms of active partnering are ongoing, and there are numerous examples of LHD collaboration with a specific organization for a specific purpose or program. However, no existing research has attempted to characterize collaboration, for the defined purpose of setting community health status priorities, between a defined population of local officials and a defined group of alternative partnering organizations. The specific aims of this study were to 1) determine the range of collaborative involvement exhibited by a study population of local public health officials, and, 2) characterize the patterns of the selection of organizations/individuals involved with LHDs in the process of setting community health status priorities.

**Methods:**

Local health department officials in North Carolina (n = 53) responded to an exploratory survey about their levels of involvement with eight types of possible collaborator organizations and individuals. Descriptive statistics and the stochastic clustering technique of Self-Organizing Maps (SOM) were used to characterize their collaboration.

**Results:**

Local health officials vary extensively in their level of collaboration with external collaborators. While the range of total involvement varies, the patterns of involvement for this specific function are relatively uniform. That is, regardless of the total level of involvement (low, medium or high), officials maintain similar hierarchical preference rankings with Community Advisory Boards and Local Boards of Health most involved and Experts and Elected Officials least involved.

**Conclusion:**

The extent and patterns of collaboration among LHDs with other community stakeholders for a specific function can be described and ultimately related to outcome measures of LHD performance.

## Background

In its landmark report of 1988, the Institute of Medicine (IOM) emphasized the importance of collaboration between public health agencies and different community stakeholders in improving community health [1]. This goal was reinforced in a 2002 IOM report that encouraged the development of collaborations representing diverse community perspectives, using community resources, and actively engaging the population in public health activities [2].

Collaboration with different community stakeholders is critical to meeting community health objectives [3-6] and typically involves diverse groups such as state and local health departments, federal and state agencies, community advisory boards, consumer rights and advocacy groups, as well as nonprofit organizations [7,8]. The primary objectives of collaboration are to leverage and expand limited resources using each partner's expertise [9-12], to reach new target populations, expand the quantity or quality of services, and prioritize community health issues using the tacit knowledge, trust, and reputation of the partners [13,14]. Collaboration can also unite diverse stakeholders and bring new perspectives on different public health issues in the communities [2,15], make decision-making more participatory and less dominated by professionals [16], as well as to overcome political resistance from those who want less governmental involvement and control [4].

While there are extensive national hospital data, there is a relative paucity of organizational data on the public health system [17]. A number of studies indicated gaps in our understanding of the role of collaboration in public health decision-making [7,18,19]. Current research calls for more inquiry on participatory decision-making in public health including types of organizations and resources involved [20]. The National Association of County and City Health Officials (NACCHO), as part of the National Profile Study, has collected information about LHD collaboration with governmental and non-governmental organizations during the period of 1990-2005. Respondents were asked to rate their level of collaboration from low to high on a 5-point scale for some general functional categories such as information exchange, work on a project or the provision of financial resources [21]. No existing research, however, has addressed the patterns of LHD collaboration with community partners in the process of priority setting.

Thus, the primary objective of this exploratory study is to understand the patterns of collaboration experienced by LHDs in determining the health priorities in their communities. Specifically, the study sought to 1) determine the range and patterns of involvement exhibited by local public health officials, and 2) identify the collaborating organizations/individuals and the extent of their involvement.

### Collaborative Involvement: Empirical Evidence from the Field

Today's organizations access expertise through formal and informal relationships as work is becoming more interdependent and collaborative in nature [22]. The IOM 2002 report concluded that collaborations are becoming more common in public health practice [2]. A number of studies suggest that collaborations are a viable strategy as LHDs are actively partnering with different entities/stakeholders in their communities to address public health issues [6,8,23-25].

The study by Mays et al. [26] reported that about 33% of the public health effort was contributed by community stakeholders and about half of the studied LHDs maintained support and communication networks with major community players. Another study found that diverse management teams were associated with higher LHD performance as they tended to have more extensive interactions in the community [15]. Erwin provided empirical evidence that LHD performance depended on how LHDs and their communities worked together [27]. Stoto et al identified a need for more effective public health collaboration including communication and coordination between LHDs and other community organizations engaged in crisis response to disease outbreaks [28]. Thus, there should be continued engagement of community stakeholders including private business and health care, conducting and monitoring their activities over time as well as across different levels (city, state) [29].

### Boards of Health

Boards of Health are often a major contributor to local public health effort. Scutchfield et al. reported that the presence of the Boards of Health was associated with higher performance of LHDs in mobilizing communities to address public health issues [7]. Mays at al. found that perceived effectiveness of public health initiatives was 14% higher in communities that had Boards of Health than in communities without them [26]. Savoia et al. found that the presence of a Board of Health was positively associated with public health emergency preparedness activities such as conducting drills, exercises and training [30].

### Elected Officials

Elected officials are often responsible for making political and public health decisions, thus, their role and influence on public health activities may be important [31]. Collaboration between elected officials and LHDs was found extremely important in making decisions regarding pharmacy sales of syringes in California [32]. Kennedy reported that higher LHD performance was associated with the perceived support by elected officials [33]. Based on the results of an empirical study, Mays et al. recommended building and maintaining a strong governmental involvement in public health systems [26].

### Community-Based Organizations

Community-based organizations are typically non-profit health and social service organizations located in the communities they service. They are familiar with their communities and, as a result, represent a crucial component of the public health system for identifying community public health issues, developing responses, and assessing results [34,35]. Cali et al. demonstrated positive outcomes of collaboration between LHDs and academic institutions and, specifically, their combined efforts at investigating and addressing local environmental health hazards [36].

### Physician and Hospital Providers

One empirical study found that medical providers were selected as major contributors to health care priority setting because of their deep understanding of community needs and expert knowledge necessary for decision-making [19]. Gadomski et al. described a number of positive initiatives resulting from collaboration between a LHD and a rural non-profit health system of community hospitals focused on preventive services [37]. Effective collaboration of LHDs with medical providers is critical because of the dynamic and quite unpredictable nature of disease outbreaks. It is important that medical providers help with disease surveillance before an outbreak occurs, and the information they provide is a critical part of outbreak identification and response [28].

### Community and Patient Representatives

A number of studies found that the general public had much to contribute to public health decision-making to supplement the contribution of health care professionals and recommended intensive community involvement in public health activities [4,19,38,39]. For instance, projects focusing on health care information needs of Hispanic populations reported that involving Hispanic community leaders in the projects from the beginning was extremely important for the success of the projects [40]. However, evaluations of public participation in decision-making and resource allocation in public health are practically non-existent despite the increasing demands for a greater representation of communities in priority setting [41]. Communities are sometimes involved late into the project or they are merely used as informants or research subjects [2].

## Methods

### Measures and sample

North Carolina's 86 LHDs include large and small health departments, public health authorities, a community health alliance and district health departments which represent multiple counties. The LHDs function with the support of the state level Division of Public Health for administrative services, generalized nursing consultation, information services provided by the State Center for Health Statistics, and accreditation. As of July 2011, 61 of the LHDs have completed an accreditation process which is based upon their capacity to perform the core functions and essential services as detailed in the National Public Health Performance Standards Program. NC LHDs have interacted with boards of health, state agencies, schools, non-profit organizations, hospitals and other organizations across a variety of issues [42]. No study, however, has addressed the nature and extent of collaboration in the process of determining community health status priorities.

The paper survey was administered to NC LHD directors or their representatives during regular monthly meetings of NC health officers in the fall of 2007. The institutional review board (IRB) of the University of North Carolina, Charlotte, approved the project protocol and survey instrument prior to data collection. Respondents consented to the use of the data, including publication of the findings, with the promise of anonymity and confidentiality. Fifty-three respondents completed the surveys, representing 62 percent of all NC local public health directors. Respondents were given a list of eight types of organizations/individuals as follows: Health Department Staff, Local Boards of Health, Patient Representatives, Community Advisory Board, Community Health Professionals (physicians, hospital administrators), Elected Officials, Community-based Organizations (United Way), and Experts. Respondents were asked to rate the level of involvement with each of the organizations/individuals on a scale from 1 (to a very little extent) to 7 (to a very great extent).

### Analysis

The analysis had three components. First, an average score was calculated for each of the eight collaborator (individuals/organizations) categories. This score was the accumulated ratings (maximum possible 7 × 53 = 371) of involvement levels (1-7) for each collaborator category divided by the number of respondents (maximum possible = 7). Second, a total involvement score was calculated for each of the 53 local public health officials. This score was the sum of all collaborator ratings (maximum possible 7 × 8 = 56). Health official involvement scores were segmented into two groups at the approximate midpoint: low involvers (n = 26) and high involvers (n = 27) and their scores were distributed among three categories of involvement extent responses (scores 1-2, scores 3-5, scores 6-7).

Third, the technique of Self-Organizing Maps (SOM) was applied to the data. SOM is a data visualization technique which reduces the dimensions in a data set and displays similarities among the data objects as groups or clusters. Essentially, we were probing to identify clusters of health officials in regard to their involvement choices and levels; and, clusters of organizations/individuals collaborators in regard to their patterns of selection by health officials. The SOM is a stochastic clustering technique that distributes data across a grid (3 × 3 cells in this case) so that neighboring instances within a grid cell are likely to be more similar to each other than to instances that are associated with other grid cells [43]. The grid cell definitions adapt over the course of many iterations (training) and every instance is compared to all grid cells and a best match identified. Over the course of iterations the learning rate, how much each instance is allowed to alter its best match, is lowered, allowing the system to settle into an equilibrium, thus producing the clusters.

## Results

### Involvement levels: Organizations/Individuals

The internal Health Department Staff received the highest average level of involvement score in the health problems priority setting (6.06) (Table 1). This inter-organizational finding was expected since it was anticipated that every local public health unit would rely heavily on internal staff resources in this process. This score also served as a benchmark for the seven intra-organizational scores for other organizations/individuals in order to provide a comparative range of values. Three organizations were clustered together with average involvement scores between 4.50 and 4.85: Community Advisory Boards, Community Health Professionals, and Local Boards of Health. Community-based Organizations (4.13) and Patient Representatives (4.02) had the next highest level of involvement. Elected Officials (3.66) and Experts (2.13) were the least involved by local public health officials in the process of determining community health status priorities.

**Table 1:** Average involvement scores

Organizations/Individuals	Mean (SD)
Health Department Staff	6.06 (1.39)
Local Board of Health	4.85 (2.02)
Community Health Professionals	4.60 (1.66)
Community Advisory Board	4.55 (1.55)
Community-based Organizations	4.13 (1.94)
Patient Representatives	4.02 (1.66)
Elected Officials	3.66 (1.56)
Experts	2.13 (1.81)

### Involvement levels: Health officers

Health official total involvement scores ranged from 12 to 51 (mean = 33.89, median = 34.0, mode = 40.00, SD = 8.19). Health officials with scores between 12 and 33 were placed in the Low involvement group and those with scores between 34 and 51 were placed in the High involvement group. Low involvers placed a higher proportion of their responses than did High involvers in the intermediate response category (scores 3-5) for four types of organizations/individuals: Health Department Staff, Local Boards of Health, Community Advisory Board, and Community Health Professionals. For three types of organizations, the Low involvers placed a lower proportion of their responses in the intermediate response category: Elected Officials, Community-based Organizations, and Experts. For Patient Representatives, both Low and High involvers placed a nearly identical percentage of responses in the intermediate category (Table 2). Overall, 50% of the Low involver responses fell into the intermediate category compared to 38% of the responses of the High involvers.

**Table 2:** Health official involvement extent scores by low and high involvers*

Organizations/individuals involved in priority setting	Involvement extent (scores 1 &2)		Involvement extent (scores 3-5)		Involvement extent (scores 6 & 7)	
	**Low-In**^**§**^	**High-In**^**‡**^	**Low-In**^**§**^	**High-In**^**‡**^	**Low-In**^**§**^	**High-In**^**‡**^
	n = 75	n = 25	n = 105	n = 83	n = 28	n = 108
Health Department Staff	2 (3%) *column * (8%) *row*	0 (0%) (0%)	11 (10%) (42%)	1 (1%) (4%)	13 (36%) (50%)	26 (24%) (96%)
Local Board of Health	4 (5%) (15%)	2 (8%) (7%)	20 (19%) (77%)	12 (14%) (44%)	2 (7%) (8%)	13 (13%) (48%)
Patient Representatives	8 (11%) (31%)	3 (12%) (11%)	15 (14%) (58%)	16 (19%) (59%)	3 (11%) (11%)	8 (7%) (30%)
Community Advisory Board	7 (9%) (27%)	2 (8%) (7%)	12 (11%) (46%)	5 (6%) (18%)	7 (25%) (27%)	20 (19%) (74%)
Community Health Professionals	7 (9%) (27%)	0 (0%) (0%)	19 (18%) (73%)	8 (10%) (30%)	0 (0%) (0%)	19 (18%) (70%)
Elected Officials	12 (16%) (46%)	2 (8%) (7%)	14 (13%) (54%)	20 (24%) (74%)	0 (0%) (0%)	5 (5%) (19%)
Community-Based Organizations	13 (17%) (50%)	0 (0%) (0%)	11 (10%) (42%)	14 (17%) (52%)	2 (7%) (8%)	13 (12%) (48%)
Experts	22 (29%) (85%)	16 (64%) (59%)	3 (3%) (11%)	7 (8%) (26%)	1 (4%) (4%)	4 (4%) (15%)

* Row and column percents do not always add to 100% due to rounding.

§ Low-involvers

‡ High-involvers

Low involvers selected low involvement scores (scores 1 and 2) more than three times as frequently as High involvers (36% vs. 11%, respectively), but High involvers selected high involvement scores (scores 6 and 7) nearly four times as frequently as Low involvers (50% vs. 13%). Not even a single High involver selected a low involvement score for Community-Based Organizations or Community Health Professionals even though Low involvers selected those response categories 50% and 27% of the time, respectively. By contrast, no Low involver selected a high involvement score for Community Health Professionals or Elected Officials, while High involvers did so 70% and 19% of the time, respectively. The high involvement responses (scores 6 and 7) represented only 13% of the Low involvers responses but 50% of the High involvers responses. These Low/High comparisons indicated a greater tendency for a generalized response among highly involved health officers than for the low involvement group. In other words, high involvement is more generalized, low involvement is more selective.

### SOMs

**Figure 1:**
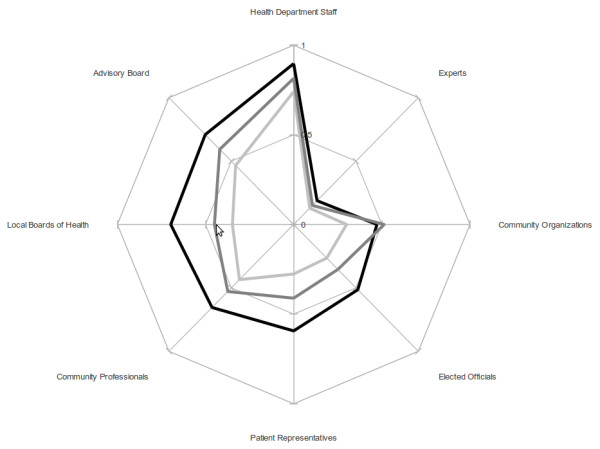
**Three involvement level clusters for 8 collaborator organizations/individuals**. Health official involvement levels: Black line high Grey line medium Light grey line low Source: Authors

The SOM technique produced three clusters of health officers whose arrangement appears to correspond generally to the overall involvement scores: upper left cell grid location; high involvement, n = 19; upper center cell grid location, medium involvement, n = 14; and, lower right cell grid location, low involvement, n = 20. The relative positions of these three clusters are displayed in Figure 1.

The three clusters exhibit a characteristic shape in regard to organization/individual collaborations. The least amount of difference among the three clusters is at the extremes; i.e. where there is the highest involvement (Health Department Staff) and the lowest involvement (Experts). For only a single organizational category (i.e. Community-based Organizations) do we see a relatively higher level of involvement for the medium involvement group than for the high involvement group, and the difference is very small. The greatest apparent distance between the high and medium involvement groups is for collaboration with the Local Board of Health.

**Figure 2:**
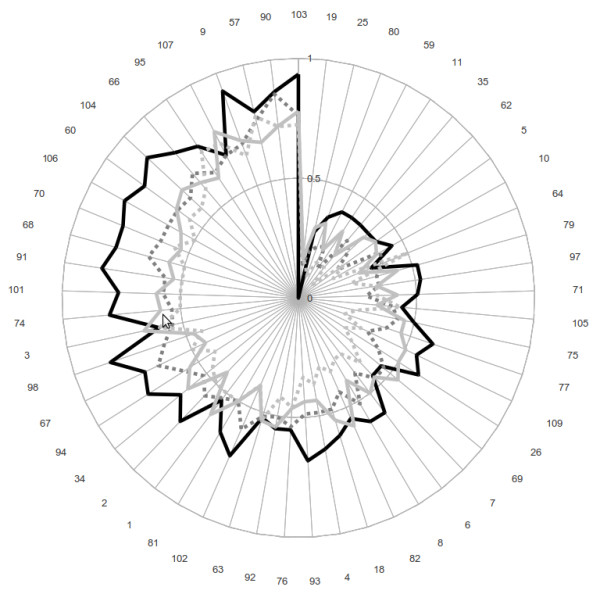
**Four collaboration clusters for 53 health officials**. Organization/individual clusters: Solid black line Advisory Board, Health Department Staff Solid light grey line Local Boards of Health Broken grey line Community Organizations/Professionals Broken light grey line Experts, Elected Officials, Patient Representatives Source: Authors

The SOM technique also produced four clusters from the eight organizational/individual categories identified in the survey (Figure 2). In this case, the data are used to identify the similarities between the organizational types and to reduce the number of types in an attempt to understand the patterns of collaboration. The four clusters were composed as follows: Community Advisory Board and Health Department Staff; Community-based Organizations and Community Health Professionals; Local Boards of Health; and, Experts, Elected Officials and Patient Representatives.

Note that individual respondents in Figure 2 are organized in a clockwise fashion from lowest to highest total involvement. For most of the range of responses, Health Department Staff and Community Advisory Board are the most prominent collaborators in priority setting. The Local Board of Health collaboration, although not quite as prominent overall, is strongest in the low medium ranges of total involvement, occasionally approaching or eclipsing that of the Health Department Staff. Community-Based Organizations and Community Health Professionals are more prominently involved with medium and high level involved public health officials. Experts, Elected Officials, and Patient Representatives were the least involved across the complete spectrum of total involvement.

## Discussion

Local public health officials exhibit a wide range of involvement with collaborators in the priority setting process, from very little involvement with any organization to extensive involvement with multiple organizations. There is a hierarchy of collaborator organizations/individuals with Community Advisory Boards/Local Boards of Health and Community Health Professionals most likely to be involved and Experts and Elected Officials least likely to be involved. Despite the variation in level of involvement among health officials, the patterns of preference for collaboration are relatively uniform; i.e. low, medium and highly involved officials maintain the same preference ranking among the eight collaborator types with only some minor variation. Officials agree most closely about both the least involved (Experts) and most involved (Health Department Staff) collaborators. Local Boards of Health appear to be most involved in the low to medium ranges of total involvement.

A number of the findings, although descriptive and without the benefit of multidimensional analyses because of data limitations, are noteworthy. The relatively low involvement with Elected Officials is somewhat surprising in that the case could be made that priority setting for resource allocation decisions may be the most important function of local government. Since LHDs in NC and many other states must often cobble together a budget derived from multiple sources, it seems the highest levels of collaborative decision making with county and city officials during this process would be advisable. Yet, only 5 of the responding officials indicated a high level of involvement with elected officials, and the SOM technique clustered Elected Officials, Experts and Patient Representatives together as the least preferred partners for collaboration. It should be noted that there may be structural characteristics which could partially account for the low level of LHD/elected officials' collaboration on health priorities such as the formal budgeting and appropriation processes involved and the prohibition of lobbying activities between government employees and elected officials.

Local Boards of Health emerged as an important influence in NC LHD priority setting. This role was further emphasized by the SOM placement of the Local Boards in their own separate cluster, indicating a unique association with local health officials. Local Boards of Health are advisory and/or governance bodies to LHDs within NC which promote the concept of citizen involvement in the local public health system as members of Local Boards of Health. Though largely voluntary and unpaid, the Boards have an obviously high profile among the LHDs for activities involving the core functions and essential services of public health. North Carolina is one of only 14 states with an Affiliated State Association of Local Boards of Health. The Local Boards of Health exhibit the highest levels of collaboration with the health officials who exhibit low to medium levels of involvement. This pattern may be an indication of the important role played by these voluntary organizations in smaller communities lacking the social capital and other resources and agencies found in more urbanized, developed areas.

The hierarchy of collaborator organizations/individuals is apparent despite the variation in the level of involvement among health officials. An important question is whether this uniform hierarchy of preferences is specific to the function, process or problem which is the object of collaborative activity? Are Experts, for example, more likely to be involved in collaborative activity with LHDs for program evaluation than seems to be the case for priority setting?

There are a number of limitations to this research which must be considered in interpreting the results. This is a study sample of 53 local public health officials in a single state (NC) and we have no comparison information for the nonrespondents. Similarly, as the result of the promise of anonymity to respondents, no information concerning health officer and community characteristics was available to use as correlates in further interpreting the extent and patterns of collaboration identified. Perhaps most importantly, due to the lack of defined health status or management outcomes, these results cannot in any way be used to characterize the performance of these health officials.

## Conclusions

These exploratory analyses have demonstrated both a range of involvement in collaboration among local health officials and a uniform pattern of collaborator preferences for one specific process; i.e. selecting community health status priority problems. Future research into the value of collaboration with community stakeholders continues to be dependent on the ability to establish valid outcome measures to determine, and empirically measure, whether the extent and pattern of collaboration results in a better selection of priorities. One approach would be to assess the "strategic congruence" of the specified priorities, or the extent to which the priorities are consistent with quantitative comparative measures of local population health status [44]. In other words, is collaboration more likely to produce a set of priorities that are evidence-based? This is an important question since many local public health functions are legally mandated or resulting from funding decisions made at the national and state levels. Similarly, community and health officer characteristics seem likely to influence the extent and nature of collaboration and are important elements to include in future research.

Ultimately, the extent and patterns of collaboration with external community partners should be informed by the degree to which that activity can be positively associated with defined community level outcomes such as desired changes in service utilization and costs and, most importantly, morbidity and mortality.

## Competing interests

The authors declare that they have no competing interests.

## Authors' contributions

JS and EAP contributed to conception and design of the study, acquisition of data, interpretation of results, drafting and reviewing the manuscript and final approval of the version to be published. CNE contributed to interpretation of results, drafting sections of the manuscript and final approval of the version to be published. JWF contributed to acquisition of data, drafting sections of the manuscript and final approval of the version to be published.
